# Normalization of human RNA-seq experiments using chimpanzee RNA as a spike-in standard

**DOI:** 10.1038/srep31923

**Published:** 2016-08-24

**Authors:** Hannah Yu, Yoonsoo Hahn, Sang-Ryoul Park, Sun-Ku Chung, Sangkyun Jeong, Inchul Yang

**Affiliations:** 1 Center for Bioanalysis, Korea Research Institute of Standards and Science (KRISS), Korea; 2 Bioanalytical Science, University of Science and Technology (UST), Korea; 3 Department of Life Science, Chung-Ang University, Korea; 4 Medical Research Division, Korea Institute of Oriental Medicine (KIOM), Korea

## Abstract

Normalization of human RNA-seq experiments employing chimpanzee RNA as a spike-in standard is reported. Human and chimpanzee RNAs exhibit single nucleotide variations (SNVs) in average 210-bp intervals. Spike-in chimpanzee RNA would behave the same as the human counterparts during the whole NGS procedures owing to the high sequence similarity. After discrimination of species origins of the NGS reads based on SNVs, the chimpanzee reads were used to read-by-read normalize biases and variations of human reads. By this approach, as many as 10,119 transcripts were simultaneously normalized for the entire NGS procedures leading to accurate and reproducible quantification of differential gene expression. In addition, incomparable data sets from different in-process degradations or from different library preparation methods were made well comparable by the normalization. Based on these results, we expect that the normalization approaches using near neighbor genomes as internal standards could be employed as a standard protocol, which will improve both accuracy and comparability of NGS results across different sample batches, laboratories and NGS platforms.

Ultra-high-throughput sequencing of RNA (RNA-seq) employing next-generation sequencing (NGS) technology is providing unchallengeable performances in transcriptome analyses^1^,^2^,^3^. However, various sequence-dependent biases and random fluctuations during RNA-seq procedures could interfere with clear and correct interpretation of the results. Therefore, an appropriate normalization of data sets from separate RNA-seq analyses is needed to achieve a better consistency and accuracy in comparisons of samples. Various normalization methods have been developed to facilitate reliable comparisons of NGS results. Normalization methods could be categorized into the quality control of experimental procedures^4^,^5^,^6^,^7^, bioinformatic processing of data based on various mathematical and statistical models^1^,^8^,^9^,^10^ or bias corrections^11^,^12^,^13^,^14^. Those normalization methods provided significantly improved but still insufficient performances. A good normalization practice for NGS-based genomic analyses requires equalization of different reaction efficiencies between separate analyses and gene-by-gene normalization of sequence-dependent biases and variations. It will be better if the normalization method is augmented by the power to simultaneously normalize all genes for modern genome scale analyses. In this point of view, previously reported methods do not fulfill all the essential requirements for an ideal normalization method.

In analytical chemistry, the isotope dilution mass spectrometry (ID-MS) is regarded as the most authentic method for quantification of organic and inorganic materials^15^,^16^. In the ID-MS strategy, an isotopic material with the same chemical formula of a target material is spiked into separate samples as an internal standard. Since an isotopic material has the same chemical property as the target material, the isotopic pair of a material will be equally influenced by various interferences and matrix effects in a given analytical condition. After discrimination in mass spectrometry, the isotopic material is used to normalize different analytical efficiencies between two separately processed samples and then cancelled out. The stable isotope labeling by amino acids in cell culture (SILAC) strategy was recently developed for normalization of modern proteomics analyses employing the concept of ID-MS^17^,^18^.

Analogically, we aimed to develop a conceptually solid normalization method for genomic analysis, which will not only enable correction of sequence-dependent biases and equalization of different reaction efficiencies resulting from physically separate analyses but also simultaneously offer genome-scale coverages. As an effort to establish such normalization method, we suggest a ‘use of a genome from a closely related species as a spike-in standard for NGS’ and present a proof of the concept by employing chimpanzee RNA as a spike-in standard for human transcriptome analysis. It has been reported that use of internal standards carrying a few discriminable sequence variations could effectively normalize inter-sample variations in amplicon sequencing leading to accurate and reproducible quantification of gene expression^19^. A similar concept, ‘normalization by discriminable spike-in standards’ was tested for human transcriptome analysis in this study. Use of a chimpanzee genome as a spike-in standard has several conceptual benefits. First, it is easy to prepare the materials to be used as standards. Chimpanzee reads will be used only for normalization of human reads and will be cancelled out in the final data. Then, tissue sources, preparation methods and qualities of spike-in chimpanzee RNA will not critically affect normalization performances due to the cancelled-out property of the chimpanzee RNA sequences. The only requirement is that the same amounts of standard materials from an identical batch should be spiked in to all samples to be compared. Second, it will allow read-by-read normalization of all individual genes that are accompanied by discriminable SNVs between humans and chimpanzees. Third, the spike-in standard will comprehensively normalize the entire NGS procedure from total RNA to data analysis. The chimpanzee RNA spiked into a human sample will represent the same reaction efficiencies as human RNA throughout the whole NGS procedure. Different efficiencies in mRNA enrichment, reverse transcription, PCR, cleanup, and sequence reading in separate reaction environments will be equalized by the spiked-in chimpanzee standard. Normalization of data with respect to sequencing throughput, GC content-dependence and amplification bias could also be simply replaced by using the spike-in standard.

## Results

### Concept of the SNV internal standard

The first consideration in selecting a spike-in standard for the RNA-seq analysis was the frequency of SNVs. Too many SNVs in a read may result in a decreased resemblance of chemical properties and reaction efficiencies between the target and the standard RNA. On the other hand, reads lacking SNVs cannot be normalized by our SNV-based strategy. In this regard, we postulated that one variation per read would be optimal for our strategy. Assuming read lengths of 150-bp by contemporary NGS platforms, one SNV per 150-bp will be the optimal frequency. SNV frequencies were estimated to be about one in 150–200-bp in chimpanzees and gorillas while much more (one in 20–50-bp) SNVs were estimated in orangutans and macaques by alignments of several ape and human mRNA sequences. Based on this estimation, we selected chimpanzee RNA as the spike-in standard for the current human transcriptome analysis.

**Figure 1:**
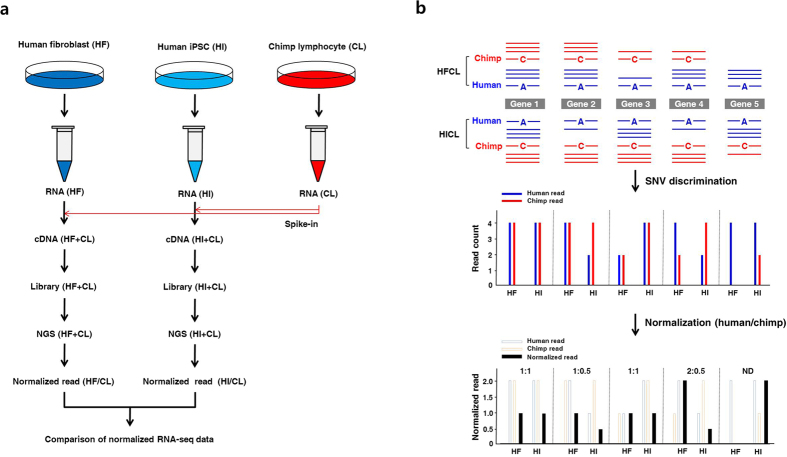
Scheme of the human transcriptome analysis using chimpanzee RNA as a spike-in standard. (**a**) Experimental workflow of the comparative transcriptome profiling of human fibroblasts and induced pluripotent stem cells using RNA from chimpanzee lymphocytes as a spike-in standard. (**b**) Normalization of human reads by chimpanzee reads in chimpanzee RNA-spiked samples (HFCL and HICL). Reads are discriminated and sorted based on SNVs in the reads. Then, human read counts are divided by chimpanzee read counts to yield read-by-read normalized human reads for the SNV loci. Then the normalized reads are compared to obtain a quantitative profile of differential gene expression between the two cell lines. ND: not determined.

Figure 1a illustrates the scheme for transcriptome analysis employing chimpanzee RNA as a spike-in standard. In this strategy, identical amounts of chimpanzee RNA are spiked as SNV-internal standards (SNV-IS) into two RNA samples to be compared. Then the spiked-in chimpanzee transcripts will behave the same as the co-existing human counterparts throughout the entire RNA-seq procedure owing to sequence similarities. After discrimination of sequences based on SNVs, commonly spiked chimpanzee RNA would be used to comprehensively equalize different analytical performances including stochastic variations, sequence-specific biases and matrix-driven interferences between separate analytical setups. For practice of SNV-normalization (Fig. 1b), chimpanzee and human reads should be first discriminated and sorted from the mixed reads data based on SNVs. Then human read count for each SNV locus should be divided by chimpanzee read count to yield a normalized read ratio for the SNV locus. Finally, these SNV-normalized reads will be compared to obtain differential expression ratio of the transcript between the two samples.

To obtain informative SNVs between humans and chimpanzees, we performed RNA-seq with pure human and chimpanzee RNAs and compared the data to discover SNVs between the two species. A total of 166,310 SNVs were identified from comparisons of 40 million common reads. Only homozygous SNVs (146,611) were chosen from the initial data because heterozygous SNVs could result in ambiguity when discriminating read sources. The homozygous SNV loci were distributed over 10,119 unique transcripts representing the frequency of 14 SNVs per transcript with an average interval of 210-bp. The selected reads representing homozygous SNVs between humans and chimpanzees were reconstructed as a reference database for discrimination of the species sources of NGS reads (http://birch.cau.ac.kr/pub/Informative-Position.txt).

### Differential gene expression analysis using chimpanzee RNA as a spike-in standard

**Figure 2:**
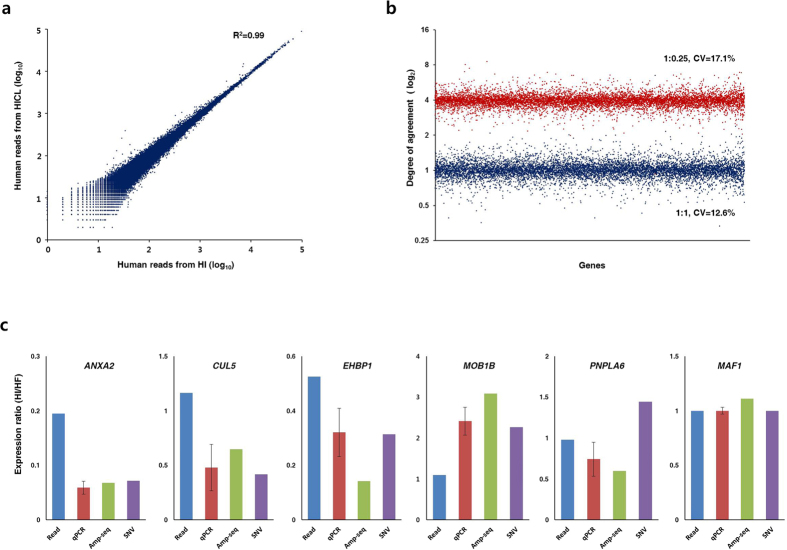
Results of the SNV-normalized transcriptome analysis. (**a**) Correlation of human reads from pure iPSC RNA (HI) and iPSC RNA spiked with chimpanzee RNA (HICL). Reads for 110,237 informative loci over the read cutoff of 40 were plotted. (**b**) Agreement profile of SNV ratios between technical duplicates of HICL with spike-in ratios of 1:1 and 1:0.25. Values for 8,656 genes over the read cutoff of 40 were plotted. (**c**) Comparison of estimates for differential gene expression between iPSCs (HI) and fibroblasts (HF) by unnormalized reads (Read), qPCR, amplicon sequencing (Amp-seq) and SNV-normalized reads (SNV). *ANXA2, CUL5, EHBP1, MOB1B*: closer agreement with estimates by SNV-normalized reads. *PNPLA6*: closer agreement with the estimate by unnormalized reads. *MAF1*: a concordantly estimated control.

As a proof of concept, the SNV-IS strategy was used to profile differential gene expression between human fibroblasts (HF) and induced pluripotent stem cells (HI). First we checked whether spike-in of the chimpanzee RNA caused any interference in RNA-seq results. It is an essential requirement that a spike-in standard should not lead to any interferences in results of original samples. Figure 2a shows the correlation between human reads from the chimpanzee RNA-spiked HI sample (HICL) and those from the pure HI sample without spike-in. The very high Pearson’s correlation coefficient of 0.99 indicated that spike-in of the chimpanzee RNA did not cause any significant interference during the RNA-seq analysis. Next, maintenance of SNV-normalized gene expression values (ratios of human reads to chimpanzee reads) between technical replicates was investigated. Low read counts generally result in a wider scattering of points in the correlation and the agreement plots, implying higher technical noises and variations^7^,^20^. Therefore, we selected genes with read counts over 40 (equivalent to an RPKM value of 2.8 by a rough calculation) for subsequent analyses to avoid misinterpretations that result from the higher technical noises for low read counts. About 86% of the genes (8,656 out of 10,116 genes) were qualified for further analysis by application of the cutoff value of 40. A good agreement (Mean = 1.01) and low coefficient of variations (CV = 12.1%) for the SNV values were observed between technical replicates of chimpanzee RNA-spiked RNA-seq experiments (Fig. 2b). In addition, normalized values were proportionally maintained when one fourth amount of chimpanzee RNA was spiked in as internal standards. The mean ‘SNV-normalized values’ from the 1-to-0.25 (human to chimpanzee) mixture was 3.97 with CV of 17.1% (Fig. 2b). The mean ratio of 3.97 is exactly inverse correlated with the spike-in ratio of 0.25, which indicates use of lesser amounts of chimpanzee RNA as spike-in standards will not result in a significant loss of analytical fidelity. This result is particularly intriguing since uses of lesser amounts of internal standards would make the SNV-normalization approach more flexible and economical to better fit analytical purposes. Based on the high degree of agreement and consistency of SNV values across replicate samples and lesser RNA-spiked samples, we expect that differential gene expression profiling based on the SNV-normalization strategy will be highly stable and accurate in most RNA-seq experiments.

Differential gene expression estimated for transcripts will not be necessarily equivalent to differential expression estimated for a specific region of the transcript due to existence of various isoforms for a gene and potential bioinformatics-driven errors during assembly to transcript information. Therefore we investigated the accuracy of estimates for differential gene expression by the SNV normalization approach not in transcript levels but for individual SNV loci. 4,264 out of 146,611 commonly detected SNV loci exhibited more than two-fold differences between the two estimation methods. The number of discordantly estimated loci was only 60 when the 85,770 loci over the read cutoff value of 40 were analyzed. To examine estimates from which methods are correct, we selected 10 discordantly and 3 concordantly estimated loci for further validation by qPCR and quantitative amplicon sequencing. Differential gene expression estimated by unnormalized read counts, SNV-normalized reads, qPCR and amplicon sequencing are compared in Fig. 2c. Results from qPCR and amplicon sequencing agreed more closely with estimates from SNV-normalized read than those from unnormalized read counts for 8 out of 10 discordant loci (Supplementary Table 1 for more information). Therefore, we concluded that the SNV-normalization method enabled a more accurate quantification of differential gene expression by equalizing various experimental artifacts and biases in separately processed RNA-seq data.

### Normalization of data sets from different in-process degradations and different library preparation methods

**Figure 3:**
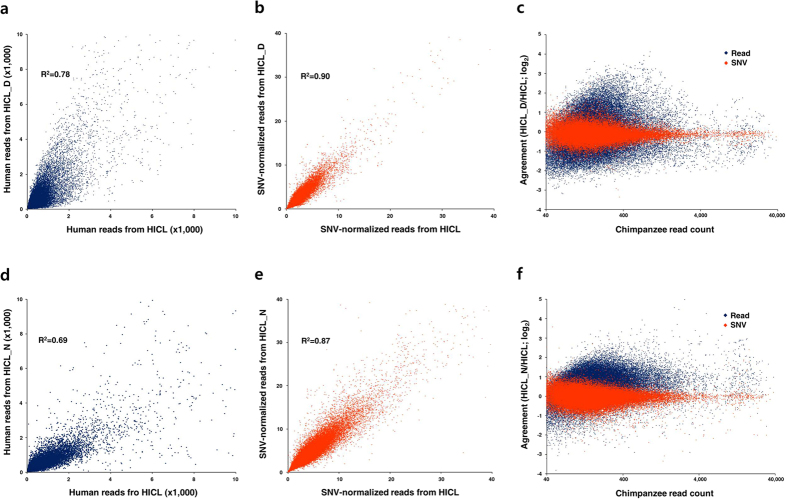
Improved comparability across degradation effects and library preparation methods. (**a**) Correlation of unnormalized reads from normally processed iPSC RNA (HICL) and in-process degraded iPSC RNA (HICL_D). Reads for 51,552 loci over the read cutoff of 40 were plotted. (**b**) Correlation of SNV-normalized reads from the HICL and HICL_D samples. (**c**) Agreement of estimates for gene expression in HICL and HICL_D samples estimated by unnormalized reads and SNV-normalized reads. (**d**) Correlation of unnormalized reads generated by TruSeq method (HICL) and NexTera method (HICL_N). Reads for 53,585 loci were plotted. (**e**) Correlation of SNV-normalized reads from the HICL and HICL_N samples. (**f**) Agreement of estimates for gene expression in HICL and HICL_N samples estimated by unnormalized reads and SNV-normalized reads.

Next, we tested our method for two artificial but probable situations where process-driven artifacts would interfere with the RNA-seq analyses. First, we compared results from a normal sample and a partially degraded sample. Identical amounts of samples from the 1:1 mixture of HI RNA and chimpanzee RNA were used for this comparison. One aliquot was subjected to library preparation after partial degradation by RNase A (HICL_D) while the other was directly processed (HICL) for library preparation. Degradation of RNA by RNase A may lead to preferential enrichment of 3′ regions of transcripts during purification of mRNAs based on oligo-dT since 5′ regions would not be captured by oligo-dT if any of the downstream regions were degraded by RNase A. Then gene expression profiles estimated from the 3′-biased reads from a degraded sample would not be highly comparable with those from relatively unbiased reads from a normal sample. As expected, unnormalized reads exhibited a very low correlation between the two data sets (R^2^ = 0.78) implying they could not be directly compared by standard methods (Fig. 3a), despite that they were from identical samples. On the contrary, SNV-normalization dramatically improved the correlation (R^2^ = 0.90, Fig. 3b), which suggested that the data were corrected comparable by the normalization. The agreement plot also showed a significant improvement of the accuracy of quantitative gene expression profiling for the degraded sample by the SNV-normalization (Fig. 3c). Comparison of estimates by unnormalized reads represented a CV of 78.4% while a CV of 28.6% was obtained by the SNV-normalization between the originally identical but differently in-process degraded samples. The results indicate that the SNV normalization could normalize and compensate various in-process variations and biases making RNA-seq experiments more consistent and better comparable. The successful normalization of data from degraded samples was possible because the chimpanzee RNA had been spiked-in before the degradation step and was influenced by the same degradation effect as coexisting human RNA.

The second approach was to compare the results from two different library preparation methods, TruSeq and NexTera. Enriched and fragmented mRNA was reverse transcribed by random hexamers in the TruSeq method while total RNA was reverse transcribed by oligo-dT and random hexamers in the NexTera method. Sequencing adaptors were attached by ligation in TruSeq while they were introduced by transposition in NexTera. Due to mechanistic differences between the two library preparation methods, substantially different read repertoires with low comparability were expected. The correlation (R^2^ = 0.69, Fig. 3d) and the agreement (CV = 59.7%, Fig. 3f) between data from the two library preparation methods were very low when analyzed based on unnormalized reads. Similarly to improvement in differently degraded samples, the correlation (R^2^ = 0.87, Fig. 3e) and agreement (CV = 26.0%, Fig. 3f) between the two library preparation methods were substantially improved by the SNV-normalization. This means spiked chimpanzee RNA behaved the same with human RNA during each library preparation and successfully normalized biases resulting from mechanistic differences of the two methods. Based on these results, we concluded that the spike-in RNA could normalize and negate various method-dependent biases and batch-dependent variations^21^, which would facilitate more consistent and accurate gene expression analysis by NGS.

### Flattening of read distributions

**Figure 4:**
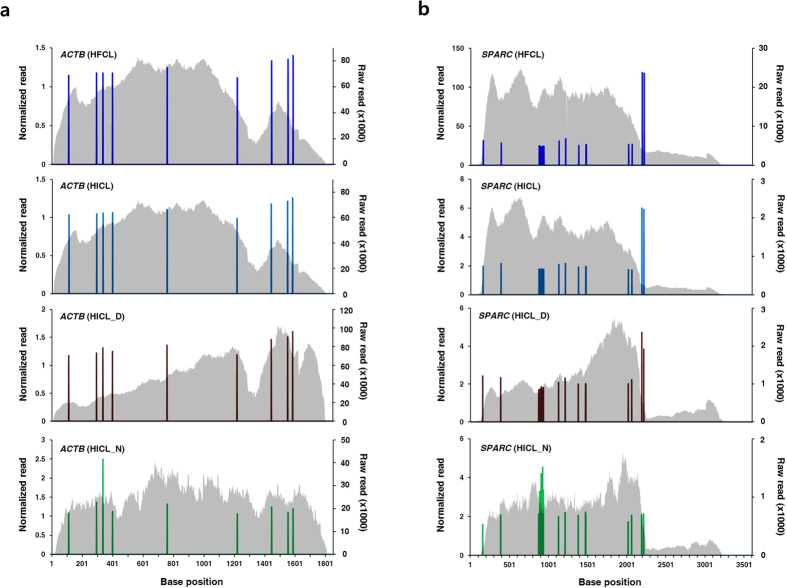
Flattening of read distributions by SNV-normalization. (**a**) Flattening of read distribution of *ACTB* gene by SNV-normalization. Gray stacks: unnormalized reads. Columns: SNV-normalized reads. (**b**) Flattening of read distribution of *SPARC* gene.

Successful normalization of RNA-seq procedures by the spike-in standard could also be monitored by changes in read distributions. Sequence-dependent biases in fragmentation of RNA, reverse transcription and PCR would result in a skewed distribution of reads over a transcript^11^,^22^. If those biases are correctly normalized by the spike-in standard, the skewed distribution pattern will become flat except for regions representing distinguishable isoforms between the two species. Figure 4a,b respectively show flattening of read distributions of *ACTB* and *SPARC* genes by SNV-normalization. Although large portions of the transcripts remain unnormalized due to lack of SNVs for the regions, the SNV-normalized reads in a transcript are of similar heights implying consistent and correct normalization of the sequenceability biases. The flattening of read distributions by the SNV-normalization is highlighted in the data from degraded samples and NexTera methods. Unnormalized read distributions from those artifactual conditions exhibit apparently different shapes compared with distributions from normal conditions. However, the SNV-normalized peaks represent almost the same distributions as those from normal conditions except for a few peaks in the NexTera data. Read distributions were also successfully flattened for most genes (http://birch.cau.ac.kr/pub/plots/).

Nonetheless, there could be a few exceptions that do not exhibit successful flattening of read distributions. For example, two SNV-normalized peaks around the 2,200-bp position of the *SPARC* transcript are distinctly higher than the other peaks (Fig. 4b). The two higher peaks seem not to be resulted from experimental artifacts since the same pattern was maintained in three results except for the NexTera data. A possible explanation for the unsuccessful flattening is absence or minimal expression of the chimpanzee counterpart for the region. Existence of complicated isoforms or differential regulation of RNA stability could also result in unbalanced transcript ratios for the region between humans and chimpanzees and subsequently unsuccessful flattening. However, those possibilities remain to be validated by further analyses such as precise exon boundary mapping and experimental examination of transcript isoform structures.

### Comparison with conventional normalization methods

**Figure 5:**
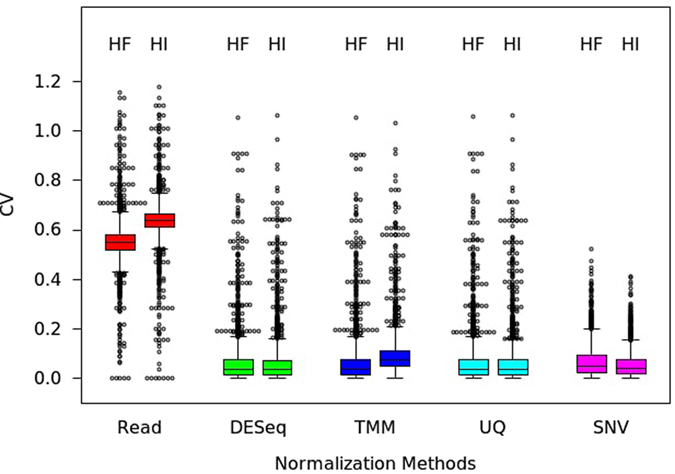
Comparison with conventional normalization methods. Box-and-whisker plots showing coefficients of variations for 2,771 housekeeping genes. Data from technical replicates were normalized either by conventional methods or the SNV method. Normalization methods are indicated below the plots.

For evaluation of normalization performance of the proposed approach, the SNV normalization method was compared with conventional methods adopting various normalization algorithms including TMM^9^, DESeq-RLE^23^, UQ^8^ for real RNA-seq data. The conventional methods were applied for normalization of technical replicate data sets of HF and HI, while the SNV method was applied for technical replicate data sets of HFCL and HICL. It was not easy to directly compare results from the SNV method with those from conventional methods, since the SNV-normalization method produces ‘human to chimpanzee ratios of transcripts’ most of which were smaller than 5, while conventional methods produce ‘normalized read counts’ that were generally greater than 100. Therefore, we indirectly evaluated those methods by comparing CV values for previously reported 2,771 housekeeping genes^24^ between the replicate data sets. Box-and-whisker plots in Fig. 5 comparatively represent CVs depending on normalization methods for technical duplicate data sets for fibroblasts and iPSCs. The SNV method exhibited similar or better normalization efficiencies as represented by lowered CVs that were comparable with values from other methods for technical replicate data sets. Therefore we concluded that the SNV method would perform similarly or better than conventional methods for normalization of RNA-seq data.

## Discussion

A potential concern about the SNV-normalization methodology could be a decrease of effective read throughput due to the occupation of sequencing capacity by non-target chimpanzee reads and SNV-deficient reads. The arithmetic decrease of read throughput was unavoidable as a trade-off for a better accuracy and comparability in realizing a novel methodology. The fraction of reads carrying at least one SNV which could be considered as effective read throughput was about 40% in this study. Read throughput will influence the capability to discover rare transcripts and the number of transcripts qualified over a noise cutoff for further analyses. With regard to rare transcripts discovery, it has been reported that discovery rates were saturated in the ranges from 10 to 40 million of 25-bp reads^1^,^20^. Then, the throughput of 22–26 million of 150-bp human reads in this study could be regarded as sufficient for rare transcripts discovery. Actually, typical human reads in this work have covered about 15,000 RefSeq NM entries exhibiting higher coverages than previously reported values^20^. Therefore, the effective read throughputs in this approach are sufficient for rare transcripts discovery as long as the transcripts are carrying SNVs.

Regarding the number of qualified transcripts, it is standard to analyze a fraction of read data over a certain noise cutoff in differential gene expression analysis^1^. The SNV-normalization method could shift the noise cutoff to a much lower point. The degree of variations in SNV-normalized reads from identical samples were markedly lower than those in unnormalized reads (Fig. 3c,f), which implied that the cutoff value guaranteeing a certain quality would be substantially lowered by the method. A lowered noise cutoff will increase the number of qualified transcripts in a given NGS data set for subsequent analyses. In addition, it has been shown that spike-in of a lesser amount of chimpanzee RNA down to 25% did not result in any significant loss of analytical performance (Fig. 2b). This observation also attenuates the concerns about the lower effective throughputs resulting from addition of an exogenous RNA as an internal standard. Based on these considerations, we concluded that the potential drawbacks from the decreased read throughput in the SNV-normalization approach will not be critically problematic and could be effectively negated by the benefit of reduced experimental variations.

Another important consideration performing SNV-normalization for NGS should be the frequency of SNVs. In theory, if more discriminating SNVs are present, more information will be obtained for normalization. The absence of SNV at a transcript will result in a failure of normalization for the transcript. On the contrary, too many SNVs in a transcript could result in different molecular properties between the target and the spike-in transcripts, which will lead to inaccurate normalization. Use of RNA from other primates such as gorilla and orangutan could be considered to increase the informative loci for normalization. The number of transcripts carrying informative SNVs and the density of discriminating SNVs in a transcript will be increased by use of those RNAs as spike-in standards. Use of spike-in standards with higher SNV frequencies will increase the effective read throughput by reducing the number of discarded reads due to lack of SNV. However, it should be first confirmed that more frequent SNVs do not affect the resemblance of the chemical properties and molecular behaviors between those spike-in standards and human transcripts. It is noteworthy that internal standards with 6 SNVs in 101-bp targets maintained similar molecular behaviors leading to accurate and stable normalization of amplicon sequencing^19^. The report indicates use of more divergent primate genomes as internal standards would lead to better normalization performances. Another approach to increase the informative loci is to use a mixture of several different cell types, which will provide a wider repertoire of expressed transcripts. Then the failure of normalization due to the absence of a transcript could be minimized. It should be noted that despite the SNV frequency was not sufficient in this study using chimpanzee RNA, average number of homozygous SNVs per transcript was measured as 14, which indicates the probability of 0 SNV-occurrence in a gene will be quite low. Then, the relatively low coverage observed in this study should be attributed more to the low complexity of chimpanzee lymphocyte transcriptome. It will be best if the tissue sources of human targets and spike-in RNA are exactly matched (for example, mixture of chimpanzee fibroblasts and iPSCs RNA as a spike-in standard for comparison of human fibroblasts and iPSCs) to solve the problem. It is also expected that the consistency and stability of normalization efficiency could be enhanced by using a cell mixture as a spike-in standard as was reported in the super-SILAC approach^25^.

In this work, we provided a proof of concept for a novel normalization methodology using chimpanzee RNA as a spike-in standard for human transcriptome analysis. The method is characterized by i) gene-by-gene normalization, ii) comprehensive normalization for the whole NGS procedures, and iii) genome-scale coverage for gene expression analysis. Consequently, accuracy and comparability were significantly improved by application of the method. The improved accuracy and comparability were not only seen in well-controlled experiments but also across different library preparation methods and different in-process degradation effects. Based on these results, we expect the SNV-based normalization methodology could be used as a standard protocol to minimize biases and variations arising from differences of samples, experimental batches, laboratories and NGS platforms.

## Materials and Methods

### Cell culture and RNA preparation

A chimpanzee lymphoblastoid cell line, EB176 (JC) was purchased from Public Health England and cultured in RPMI1540 containing 15% FBS. A human foreskin fibroblast cell line purchased from System Biosciences was cultured in DMEM containing 15% FBS. Human iPSCs were derived from a human immortalized lymphoblastoid cell line under the approval of Institutional Review Board (IRB) committee in Korea Institute of Oriental Medicine (I-1210/002/002-02). The IRB approval defined uses of previously reported experimental protocols^26^,^27^ and conformity to general guidelines on biosafety and bioethics. Derived iPSCs were maintained under mTesR1 conditional medium (Stemcell Technologies) with 0.5 mM sodium butylate (Sigma) and 25 μM SB431542 (Sigma). Total RNA was extracted using Direct-Zol (Zymo Research). Five μg of chimpanzee RNAs from an identical batch were spiked into 5 μg of human RNAs from fibroblasts and iPSCs. Two μg aliquots of the mixed RNA were subjected to preparation of library for NGS.

### Preparation of NGS library

Normal NGS library was constructed using TruSeq RNA Sample Preparation Kit v2 (Illumina) following the manufacturer’s instruction starting from 2 μg of total RNA. NexTera library was constructed following the protocol in TotalScript RNA-Seq Kit (Epicentre) with a few modifications. A mixture of 2 μg of human and chimpanzee total RNA was reverse transcribed by combined primers of 50 pmol oligo-dT and 17 ng random hexamers using Superscript III (Life Technologies). After second strand synthesis using the second strand master mix in the TruSeq RNA Sample Preparation Kit, cDNA was subjected to the library preparation procedure using NexTera DNA Sample Preparation Kit (Illumina). For preparation of partially degraded RNA, 2 μg of human and chimpanzee total RNA mixture was treated with bead-bound RNase A. The bead-bound RNase was prepared by attaching 0.1 μg of RNase A (Sigma R4642) to carboxyl magnetic beads (Spherotech CM-50-10) using the 1-ethyl-3-(3-dimethylaminopropyl) carbodiimide (Sigma) chemistry. The RNase-attached beads were washed and resuspended in 100 μL of 0.5 X PBS buffer (pH 7.0). Five μL of the RNA-attached beads solution was used for partial degradation of the RNA in 0.2 X PBS buffer for 3 minutes at 25 °C. About 70% of the 28S rRNA band was degraded and smeared by this partial digestion condition. To prevent further degradation 10 unit of RNaseOUT (Life Technologies) was added to the RNA solution separated from the beads. Then the RNA solution was directly subjected to the mRNA isolation and library preparation procedure using TruSeq RNA Sample Preparation Kit. RNA-seq was performed on HiSeq 2500 (Illumina) platform with a paired-end read length of 150-bp.

### qPCR and amplicon sequencing

Primers for qPCR and amplicon sequencing are listed in Supplementary Table 1. RNA from fibroblasts and iPSCs were reverse transcribed by random hexamers using Superscript III and the resulting first cDNA were used as templates for qPCR. qPCR were performed under standard two-step cycling conditions using TaqMan Universal PCR master mix (Life Technologies) on StepOne Plus platform (Life Technologies). One tenth of the double strand cDNA from the TruSeq procedure for library preparation were saved and used as templates for amplicon sequencing. Since the templates for amplicon sequencing were obtained from the RNA-seq procedures after spike-in of chimpanzee RNA, they represent both human and chimpanzee transcripts. Amplicons were prepared by standard two-step PCR using SYBR Premix ExTaq (Takara). Purified amplicons were subjected to library preparation by TruSeq DNA Sample Preparation Kit (Illumina). Amplicon sequencing was performed on MiSeq (Illumina) platform with a paired-end read length of 250-bp. Human and chimpanzee reads were discriminated based on SNVs in the amplicon and normalized gene expression levels were calculated by dividing the human reads with the chimpanzee reads.

### Bioinformatics

#### Adapter trimming

Reads were sorted according to the sample source, each of which had different barcode sequence. Forward- and reverse-end reads were separately stored as ‘R1’ and ‘R2’ files, respectively. The barcode sequence was removed from the 5′ end of the read. The 3′ end adapters and trailing sequences were trimmed using reaper (version 14-020) available at http://www.ebi.ac.uk/~stijn/reaper/src 28. The command line was “reaper –basename OUTPUT_FILE_BASENAME –i INPUT_FILE_FASTQ –geom no-bc -3pa ADAPTER_SEQ –tabu -” where ADAPTER_SEQ was a 22-nt sequence derived from the TruSeq or NexTera adapters and one of the following: TruSeq R1, GATCGGAAGAGCACACGTCTGA; TruSeq R2, AGATCGGAAGAGCGTCGTGTAG; NexTera R1, CTGTCTCTTATACACATCTCCG; NexTera R2, CTGTCTCTTATACACATCTGAC.

#### Human RefSeq mRNA database

The human RefSeq mRNA sequences were downloaded from the National Center for Biotechnology Information (NCBI) web site (ftp://ftp.ncbi.nlm.nih.gov/refseq/H_sapiens/mRNA_Prot) in February 11, 2014. The well-annotated RefSeq mRNAs of which accession number starts with ‘NM’ were extracted. To reduce database complexity and computational complication in this pilot analysis, only one transcript per gene, the one that appeared first in the data, was selected. The human mRNA sequences were indexed for BWA and SAMtools.

#### RNA-seq mapping

Reads were aligned to the human RefSeq mRNAs using ‘BWA-MEM’ algorithm implemented in Burrows-Wheeler Aligner (BWA 0.7.9a-r786) which was available at http://bio-bwa.sourceforge.net 29. The resulting SAM files were filtered to select reads that mapped to only one location while converting them to BAM files using SAMtools version 0.1.19-44428cd available at http://samtools.sourceforge.net 30. The command line was “samtools view –S SAM_FILE –q 1 –b –o BAM_FILE”. The BAM files then were sorted and indexed for further analyses. The percentages of uniquely mapped reads ranged from about 80% to 88%.

#### Identification of informative SNVs

To identify informative positions showing single nucleotide difference between human and chimpanzee mRNAs, reads from pure human fibroblasts (HF), human iPSCs (HI), and chimpanzee lymphocytes (CL) were comparatively analyzed. To increase the read depth, R1 and R2 reads for each of HF, HI, and CL reads were merged. SAMtools and BCFtools were used to call variations in HF, HI, and CL data compared to human RefSeq mRNAs. The command line was “samtools mpileup –d 1000000 –u –f FASTA_FILE BAM_FILE | bcftools view –bvcg - > BCF_FILE” where FASTA_FILE was the human RefSeq mRNA data. In order to increase the depth reported by SAMtools, the maximum depth count was increased from 8,000 to 1,000,000 (the 438th line of the ‘bam_pileup.c’). Each of the three BCF files was filtered to collect variations supported by at least 10 reads. The command line was “bcftools view BCF_FILE | vcfutils.pl varFilter –d 10 –D 1000000 > VCF_FILE”. The three VCF files were analyzed to collect informative positions that passed the following conditions: (i) read depth was at least 10 in all the three reads; (ii) each of HF, HI, and CL was homozygous (to allow sequencing error up to 10%, positions where the major allele was at least 90% were kept); (iii) HF and HI had the same sequence but CL had a different sequence. There were 146,611 informative positions identified from 10,119 transcripts. The informative positions were stored as a BED file.

#### Quantitation of transcripts

To quantify human and chimpanzee transcripts in a mixed sample, numbers of human and chimpanzee alleles at each informative position of a transcript were counted. First, read sequences at each informative position were extracted; the command line was “samtools mpileup –d 1000000 –f FASTA_FILE –l BED_FILE BAM_FILE” where BED_FILE contained coordinates of the informative positions. The output files were parsed to distinguish and count human and chimpanzee alleles at each position. Ratios of the human to chimpanzee allele counts were calculated for each informative position. The R1 and R2 reads were analyzed separately and showed almost the same results.

#### Comparison with conventional normalization methods

For transcriptome analysis by conventional methods, the RNA-Seq reads were mapped to the reference human genome (hg 19) using TopHat^3^. The reference genome sequence and annotation data were downloaded from the UCSC website (http://genome.uscs.edu). The transcript counts were calculated using HTSeq (http://www-huber.embl.de/HTSeq/doc/overview.html). Three conventional normalization methods; upper quartile (UQ)^8^, DESeq-relative log expression (RLE)^23^, and EdgeR-trimmed mean of M-values (TMM)^9^ were compared with the SNV-normalization method. For SNV-normalization, reads for all SNV loci in a transcript were discriminatively summed to derive total human SNV reads and total chimpanzee SNV reads for the transcript. Then, the summed human reads were divided by summed chimpanzee reads to derive an SNV ratio for the transcript.

## Additional Information

**Accession Codes**: The sequencing data from this study have been submitted to the NCBI Sequence Read Archive (SRA) under accession no. SRP046066.

**How to cite this article**: Yu, H. *et al.* Normalization of human RNA-seq experiments using chimpanzee RNA as a spike-in standard. *Sci. Rep.* **6**, 31923; doi: 10.1038/srep31923 (2016).

## Supplementary Material

Supplementary Information
