# Supplementary material for: Normalization of human RNA-seq experiments using chimpanzee RNA as a spike-in standard
Source: Sci Rep. 2016 Aug 24;6:31923. doi: 10.1038/srep31923 (PMC4995400; doi:10.1038/srep31923)
Supplement: Supplementary Information [file srep31923-s1.pdf]

Supplementary information

Normalization of human RNA-seq experiments using chimpanzee RNA as a spike-in standard

Yu, Hannah[1,2,¶], Hahn, Yoonsoo[3,¶], Park, Sang-Ryoul[1,2], Chung, Sun-Ku[4], Jeong, Sangkyun[4], and Yang, Inchul[1,2,*]

Supplementary Table 1. Validation of differential gene expression. Fold changes of gene expression between iPSCs and fibroblasts estimated by throughput-normalized read counts (Read), SNV-normalized read ratios (SNV), amplicon-sequencing (Amp-seq) and qPCR are compared. Genetic locus and sequences of primers and probes for qPCR are described for each gene. Numbers in primers represent positions of nucleotides in the given RefSeq sequences. qPCRs were performed by three independent experiments each with triplicate reactions.

Supplementary Table 1

RefSeq ID	Gene	SNV position	Primers and probes	Differential gene expression(HI/HF)				
				Raw read	SNV	Amp. Seq.	qPCR	SD(qPCR)
NM_001136015	*ANXA2*	631	ANXA_526: AACCGACGAGGACTCTCTCA<br>ANXA_713: CAGAGCCATCCTCTGCTCTT<br>ANXA_hyb: CCAGGAGCTGCAGGAAATTA	0.21	0.09	0.07	0.06	0.01
NM_003478	*CUL5*	411	CUL5_317: CACAAAGGCTAATCCGAAGG<br>CUL5_475: CTACATGGTCACCGGAGCTT<br>CUL5_hyb: AGTCGATGCTTCCTCTTCCA	1.16	0.41	0.65	0.48	0.21
NM_015252	*EHBP1*	201	EHBP_178: GGGATTAGAGCCCTCCAGAA<br>EHBP_361: AATGATGCTTGGGGTCCATA<br>EHBP_hyb: GTGTTCTTGACTGGGCCATT	0.62	0.24	0.14	0.32	0.09
NM_020746	*MAVS*	5578	MAVS_5550: CTGATCCCAGCACTTTTCAA<br>MAVS_5719: GCCACACTCTCAACACAGGA<br>MAVS_hyb: TGGCCAGTGTCAGATCTCAG	0.85	1.90	1.26	0.37	0.15
NM_001244766	*MOB1B*	5710	MOB_5594: CCGTGGAAATCACTGTTGTT<br>MOB_5764: TGTGATCAGCCTATTAGGAAAGC<br>MOB_hyb: TTCTGACCACAGTTGATTCTGAA	0.96	2.13	3.08	2.41	0.34
NM_002577	*PAK2*	5244	PAK_5035: TGTCCAGCTGCGTACAGTCT<br>PAK_5303: CACTGACATTTCACTAGATGTGGA<br>PAK_hyb: ACCTGTGCCTCTAACAAGCG	0.72	2.17	4.65	1.48	0.57
NM_001166111	*PNPLA6*	2842	PNPL_2774: AGGAGGATGCACACCGTATC<br>PNPL_3018: GCTGCGCATATTTAGCCACT<br>PNPL_hyb: AGCAGCTAGTCCTGCTCCAC	1.03	1.87	0.60	0.74	0.21
NM_053275	*RPLP0*	1219	RPLP_1010: CCTTCCCACTTGCTGAAAAG<br>RPLP_1249: AAGCCTTTATTTCCTTGTTTTGC<br>RPLP_hyb: GCTGATCCATCTGCCTTTGT	0.97	1.84	1.55	2.25	0.85

NM_003118	*SPARC*	926	SPARC_854: CCGGGACTTCGAGAAGAACT<br>SPARC_1058: CTCATCCAGGGCGATGTACT<br>SPARC_hyb: AGACCTGTGACCTGGACAATG	0.14	0.06	0.08	0.06	0.00
NM_003380	*VIM*	836	VIM_754: TCGCCAACTACATCGACAAG<br>VIM_996: GAAGCATCTCCTCCTGCAAT<br>VIM_hyb: CTCTACGAGGAGGAGATGCG	0.12	0.02	0.04	0.01	0.00
NM_021807	*EXOC4*	4071	EXO_3919: GTGGGCACTTACCATGTTCC<br>EXO_4128: GGTACAAAATCCCCCACCTT<br>EXO_hyb: TTGAAAACTCGTGGAAAGGG	1.11	1.04	1.06	1.00	0.08
NM_032272	*MAF1*	1312	MAF_1181: AGGAGGTGGAGGAAGAAAGC<br>MAF_1425: CAAAGGGCTAGGCAGGTATG<br>MAF_hyb: TATTTGATGAGGAGGAGCCG	1.00	1.00	1.11	1.00	0.05
NM_003486	*SLC7A5*	2909	SLC7_2766: GCACCCCTTGTTTCTGTTGT<br>SLC7_2998: AGTGACGCTGGACCACTTG<br>SLC7_hyb: ACCCCCAGAACAAGAAGACC	0.95	0.96	1.24	1.00	0.17
